# Contrastive Learning vs. Self-Learning vs. Deformable Data Augmentation in Semantic Segmentation of Medical Images

**DOI:** 10.1007/s10278-024-01159-x

**Published:** 2024-06-10

**Authors:** Hossein Arabi, Habib Zaidi

**Affiliations:** 1 Division of Nuclear Medicine and Molecular Imaging, Geneva University Hospital, CH-1211 Geneva 4, Switzerland; 2 Department of Nuclear Medicine and Molecular Imaging, University of Groningen, University Medical Center Groningen, 9700 RB Groningen, Netherlands; 3 Department of Nuclear Medicine, University of Southern Denmark, DK-500 Odense, Denmark; 4 University Research and Innovation Center, Óbuda University, Budapest, Hungary

**Keywords:** Deep learning, Segmentation, Contrastive-learning, Self-learning, Data augmentation

## Abstract

To develop a robust segmentation model, encoding the underlying features/structures of the input data is essential to discriminate the target structure from the background. To enrich the extracted feature maps, contrastive learning and self-learning techniques are employed, particularly when the size of the training dataset is limited. In this work, we set out to investigate the impact of contrastive learning and self-learning on the performance of the deep learning-based semantic segmentation. To this end, three different datasets were employed used for brain tumor and hippocampus delineation from MR images (BraTS and Decathlon datasets, respectively) and kidney segmentation from CT images (Decathlon dataset). Since data augmentation techniques are also aimed at enhancing the performance of deep learning methods, a deformable data augmentation technique was proposed and compared with contrastive learning and self-learning frameworks. The segmentation accuracy for the three datasets was assessed with and without applying data augmentation, contrastive learning, and self-learning to individually investigate the impact of these techniques. The self-learning and deformable data augmentation techniques exhibited comparable performance with Dice indices of 0.913 ± 0.030 and 0.920 ± 0.022 for kidney segmentation, 0.890 ± 0.035 and 0.898 ± 0.027 for hippocampus segmentation, and 0.891 ± 0.045 and 0.897 ± 0.040 for lesion segmentation, respectively. These two approaches significantly outperformed the contrastive learning and the original model with Dice indices of 0.871 ± 0.039 and 0.868 ± 0.042 for kidney segmentation, 0.872 ± 0.045 and 0.865 ± 0.048 for hippocampus segmentation, and 0.870 ± 0.049 and 0.860 ± 0.058 for lesion segmentation, respectively. The combination of self-learning with deformable data augmentation led to a robust segmentation model with no outliers in the outcomes. This work demonstrated the beneficial impact of self-learning and deformable data augmentation on organ and lesion segmentation, where no additional training datasets are needed.

## Introduction

Cumulative learning could be achieved in machine learning techniques through transfer learning, wherein a pre-trained model is employed to develop a dedicated model for a new task with an associated dataset for fine-tuning [1]. Normally, early layers of deep learning architectures capture basic features/structures such as edges, while complex features/structures are decoded by later layers. Hence, only the trainable parameters of the later layers are modified within transfer learning [2]. The similarities between the pre-trained model and the target task determine the effectiveness of transfer learning [3].

Regarding the scarcity of large labeled data in medical imaging (for the segmentation task), transfer learning or data augmentation techniques are considered effective strategies to enhance the performance of deep learning-based solutions [4–6]. Transfer learning techniques tend to rely on the inter-domain or inter-task commonality to boost the performance and/or robustness of existing machine learning solutions. Conversely, data augmentation techniques tend to generate realistic training samples (relying on the available dataset for the target task) to enrich the feature space and the overall performance of the model [7].

In addition to data augmentation techniques (applied on the training datasets), contrastive learning and self-learning approaches have been proposed/applied to improve model training through conducting sort of transfer learning using the same training dataset/samples [8–10]. Self-learning techniques enable the machine learning models to enhance the efficiency of training through obtaining supervisory signals from the training dataset. In general, self-learning approaches rely on the estimation/prediction of hidden and/or unobserved parts/properties of the input data from the rest of the input data [11]. For instance, parts of the input image could be removed and then predicted from the remaining image (based on object completion concept [12]) to learn the underlying structures/properties of the data [13, 14]. Similarly, contrastive learning techniques tend to learn the discrimination between similar and dissimilar representations, extracted/generated from the training dataset to capture the underlying discriminative features of the input data [15]. The representation samples could be generated through orientating, cropping, and deforming the input data [16, 17].

Overall, self-supervision is considered a promising framework for medical image analysis since fully annotated task-specific datasets are rather scarce, while large unannotated datasets are readily available (i.e., organ segmentation from CT images). Large unannotated datasets could be explored by the deep learning models through self-learning and contrastive learning techniques to decode primary properties/underlying structures of the data involving large variability in patients/anatomies/diseases [18–21].

Data augmentation, self-learning, and contrastive learning are recognized for their potential in enhancing medical image segmentation, particularly in scenarios with limited annotated data. However, despite their promise, significant gaps persist in this field. One prominent gap is the need for a comprehensive comparative study that systematically evaluates the effectiveness of these methods in improving segmentation accuracy. While individual studies have explored the benefits of data augmentation, self-learning, and contrastive learning, there is a lack of direct comparison between these techniques. Such a comparative study is crucial for understanding the strengths and limitations of each approach and identifying the most suitable method for specific medical imaging tasks [22].

In this work, we set out to investigate the impact of data augmentation and contrastive learning and self-learning on the performance of the deep learning-based semantic segmentation. To this end, three different datasets were employed, including brain tumor and hippocampus delineation from MR images and kidney segmentation from CT images [23]. Since data augmentation techniques also aim at enhancing the performance of deep learning methods (through generating synthetic training samples), the performance of the contrastive learning and self-learning technique was compared to a data augmentation method for the three datasets. The segmentation accuracy for the three datasets was assessed with and without applying data augmentation, contrastive learning, and self-learning to individually investigate the effectiveness of these techniques.

## Materials and Methods

### Imaging Datasets

Three datasets were exploited to investigate the effectiveness of data augmentation, contrastive learning, and self-learning on the performance of deep learning-based semantic segmentation. The first dataset belonged to the KiTS19 challenge for kidney and kidney tumor delineation from CT images [24]. This dataset is composed of 210 subjects with CT images acquired between 2010 and 2018 at the University of Minnesota Medical Center, USA. The CT images has a size of 512 × 512 voxels. Left and right kidneys were manually delineated on CT images (regarded as reference) in the training dataset [25]. Left and right kidney masks were cropped from the original CT images and were then rotated to form a single database containing both left and right kidneys. For the training of the deep learning models, CT images were normalized to a range between 0 and 1.

The second dataset belonged to the Decathlon medical segmentation challenge (http://medicaldecathlon.com) containing 260 subjects who underwent T1-weighted MPRAGE MR imaging using 860/3.7/8.0 ms, TI/TE/TR, and 1.0 mm^3^ voxel size. The hippocampus head and body were manually delineated on MR images. A single whole hippocampus mask was created by merging the body and head masks for the training of the models [26].

The third dataset belonged to the BraTS 2021 segmentation challenge from multiple MR images [27–29]. There are four different MR sequences in this dataset including native and post-contrast T1-weighted, T2 fluid attenuated inversion recovery (T2-flair), and T2-weighted. The entire dataset was manually segmented (by one to four observers) to the Gd-enhanced tumor, the peritumoral edematous/invaded tissue, and the necrotic tumor core. For this work, only T2-flair image was employed to examine the different deep learning training frameworks. Moreover, the three tumor tissues were merged to create a single mask for the whole tumor (WT) [30, 31], based on which the segmentation accuracy of different training frameworks was assessed. Prior to the implementation of the segmentation approaches, MR images were pre-processed by N4 bias field correction and noise suppression [32, 33]. Moreover, MR intensity normalization was conducted using intensity levels at 90% of the cumulative histogram.

### Training Strategies

#### Contrastive Learning

Contrastive learning is a solution to address the issue of data sparsity. In this paradigm, the model (or certain compartments of the model) is pre-trained on the same dataset, but for a proxy task. The proxy task requires that the model learn/encode the semantics and underlying features of the data/images [34]. The proxy task assigned to train the model was to classify whether the extracted image patches belong to the same subject. To this end, the encoding part of the network should learn the distinctive features and underlying structure of the data. To this end, the input images were resampled into 3 × 3 cm (patches of 3 × 3 cm) to contain sufficient data to make a distinction between the matched samples. 3 × 3 cm patches (voxel size = 1 mm) were extracted from the input images using the reference mask. This approach enabled to obtain more samples both from within and around the peripheries of the target region, enhancing the dataset’s ability to represent the areas of interest more accurately.

**Fig. 1:**
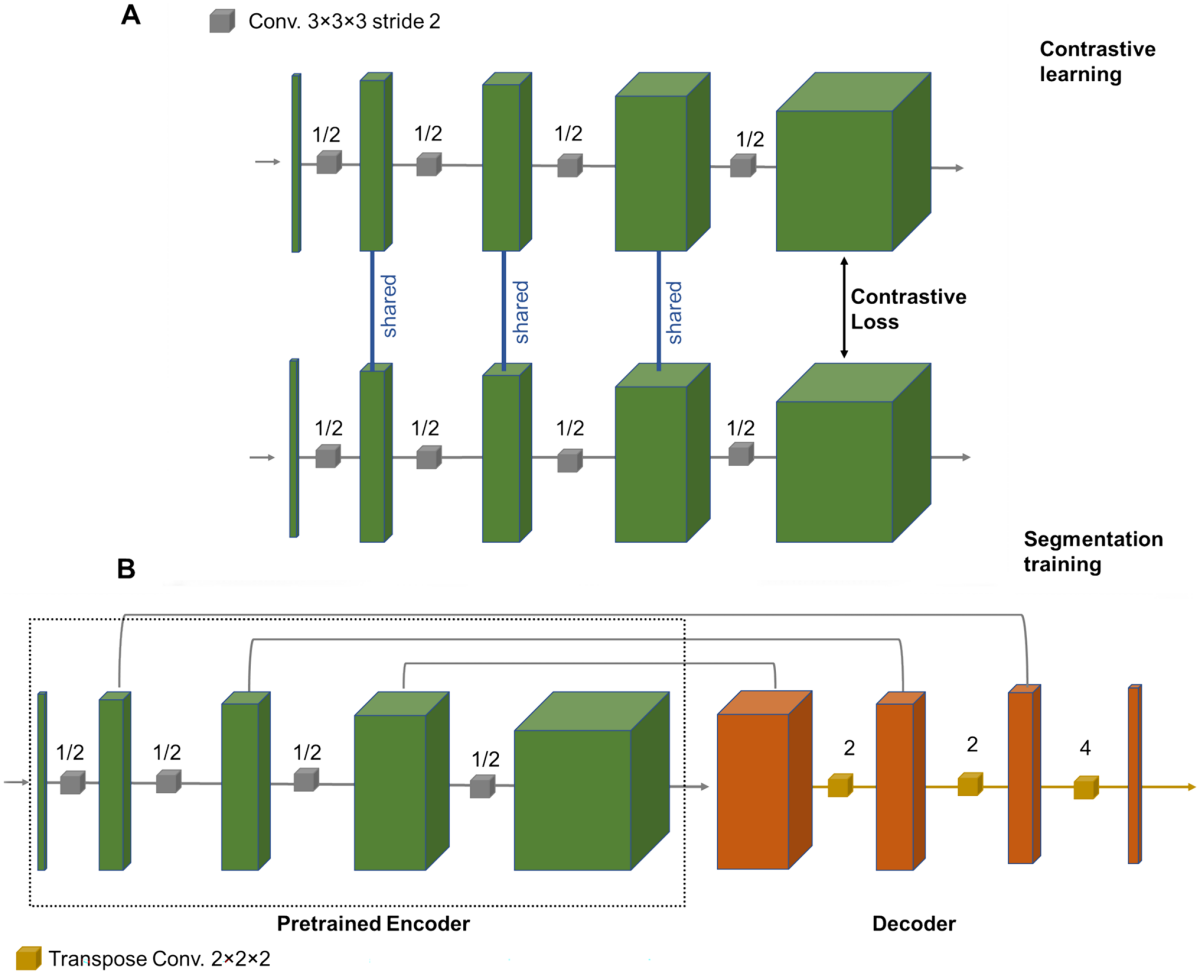
Structure of contrastive learning. **A** The encoder compartment is trained for a proxy task. **B** The trained encoder compartment is inserted into the final model for the target segmentation task

Figure 1 depicts the structure of contrastive learning, wherein in the first learning process (panel A) the encoder compartment of the model is trained to discriminate the matched patches of the image. The encoder compartment is then coupled to a decoder compartment to form the entire model (panel B). The model is then trained for the target task, which is organ/lesion segmentation in this study.

The proxy task for the training of the encoder compartment is to identify the patches of images for the same subject. This contrastive learning model, composed of two compartments, wherein the trainable weights of the encoder component are shared between proxy and target task training. In the proxy training (Fig. 1A), two branches of the encoder network are given random patches of subjects, and a label 1 or 0 is assigned if the patches are from the same or different subjects, respectively. The training for the proxy task is performed using a contrastive loss defined in Eq. 1.

1 $${Loss}_{contrastive}={\sum }_{i=0}^{I}L.{Dis}^{2}+\left(1-L\right).{Dis}^{2}$$

In Eq. (1),* L* denotes the label and $Dis={\Vert {p}_{1}- {p}_{2}\Vert }^{2}$ is the distance between *p*_*1*_ and *p*_*2*_ parameters embedded into the last layer of the contrastive learning for each branch of the encoder compartment (Fig. 1A). This contrastive loss tends to minimize the distance between *p*_*1*_ and *p*_*2*_ parameters when the label is 1 (and maximizing when the label is 0). Once contrastive training was accomplished (for the proxy task), one of the encoder branches is inserted into the final model (Fig. 1B) to perform the target training. Owing to the fact that the training weights are shared between the two branches of the contrastive learning network (Fig. 1A), any of these branches could be employed in the final model. For the target training, the encoder compartment of the final model, which is already pre-trained, is linked to a decoder network with randomly initialized weights. For this network, a hybrid loss function based on a sum of Dice and cross-entropy loss led to peak performance. We did not freeze the trainable weights of the encoder compartment with the target training in order to allow the network to be fine-tuned for the target task.

#### Self-Learning

**Fig. 2:**
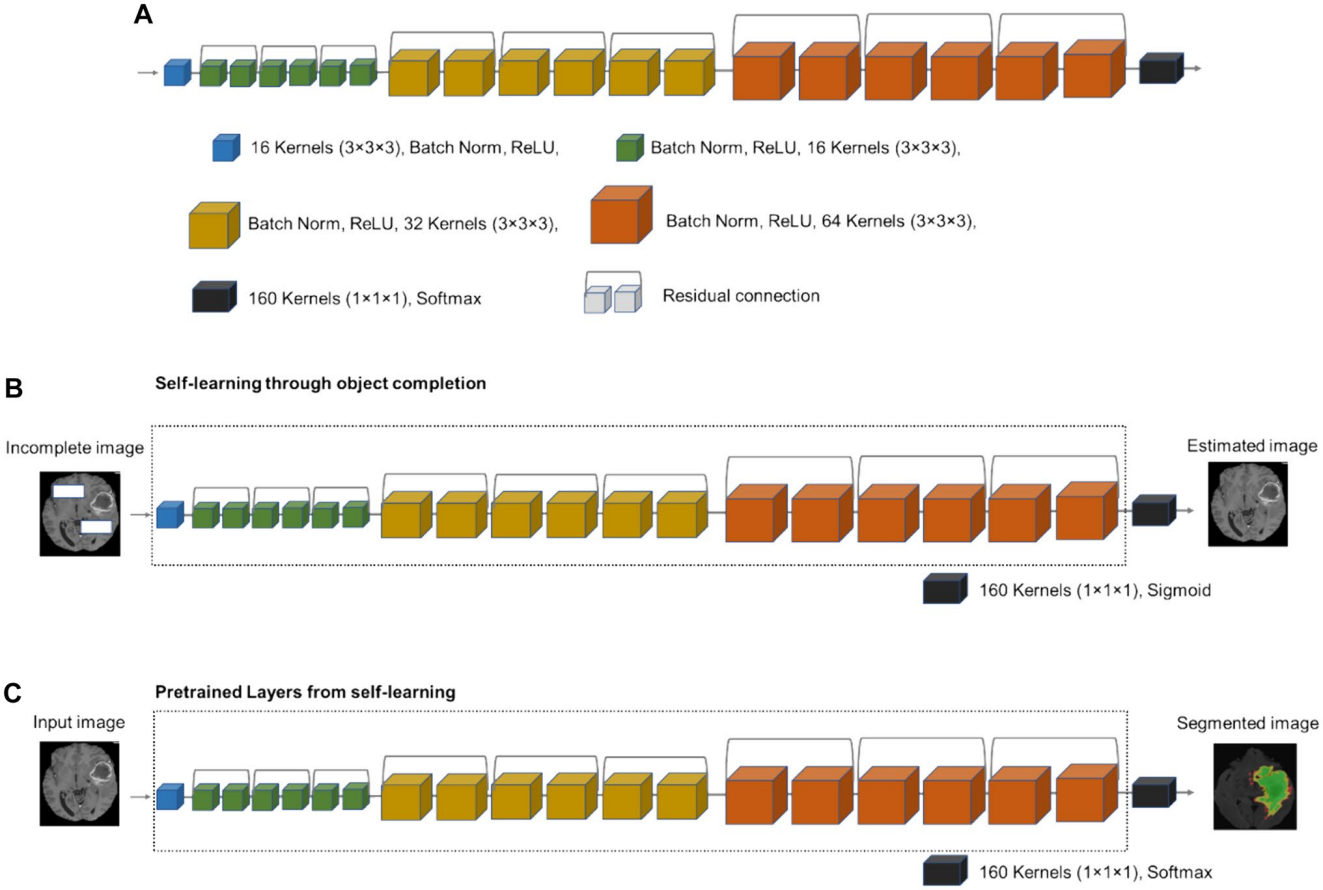
Self-learning procedure. **A** The structure of the residual neural network. **B** Pretraining of the model using a proxy task (object completion). **C** Fine-tuning the model for the target task (segmentation)

**Fig. 3:**
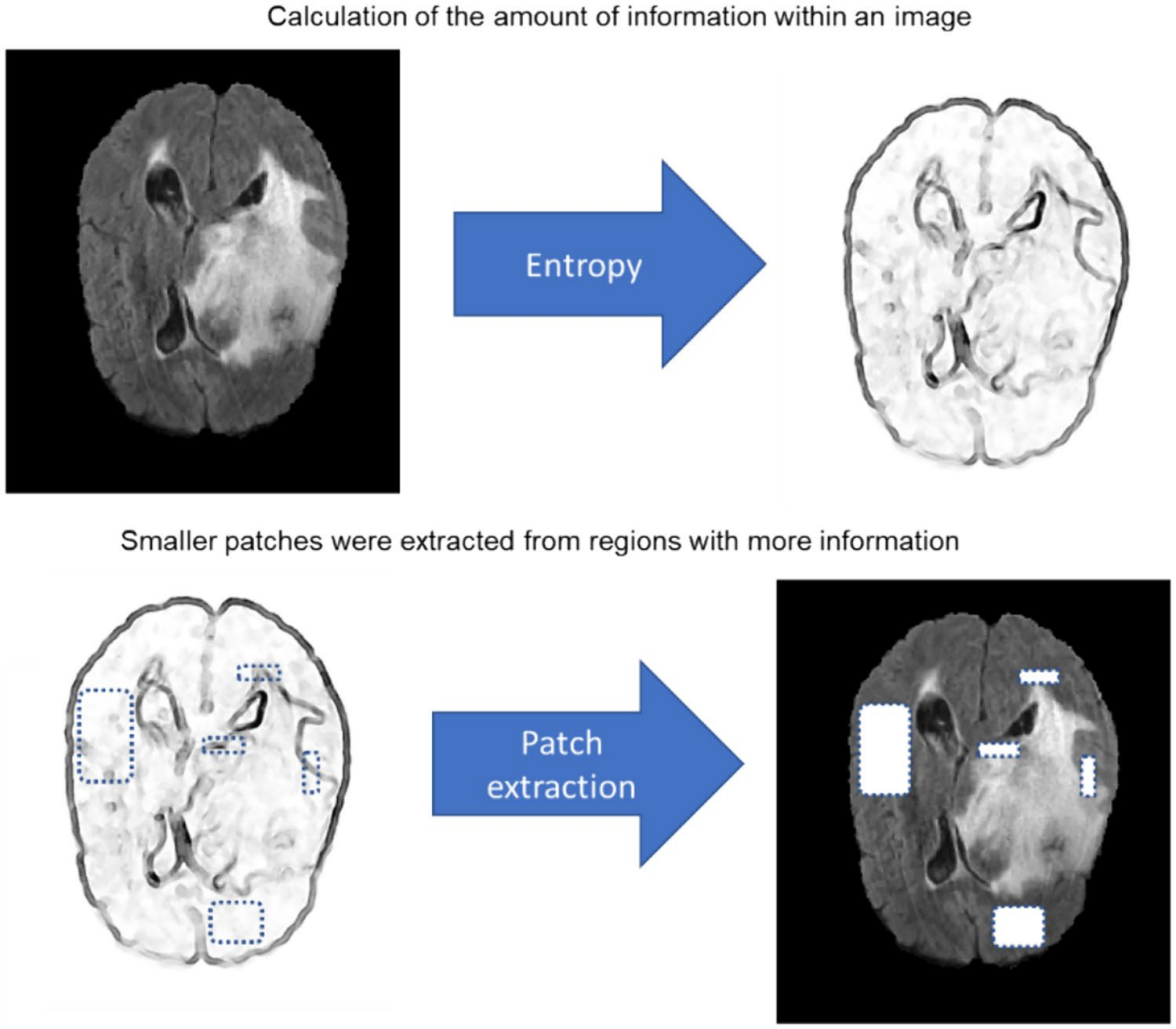
Patch selection for the self-learning task. The entropy of the input data is calculated based on which a larger number, but smaller samples are taken from regions with more information and vice-versa

For self-learning, an image inpainting (object completion) task was selected to pretrain the network to decode the underlying features/structures of the input data. To this end, a residual neural network with twenty layers (Fig. 2A) was developed to predict/estimate the missing patches of the image in the input data (Fig. 2B) [35]. Patches of voxels were eliminated from the input data to be predicted by the residual network. In order to efficiently conduct the process of image inpainting, the entropy of the input data was first calculated, which indicates the levels of information within the input image (Fig. 3). Thereafter, larger patches of voxels were selected from regions bearing a low amount of information, while smaller patches were taken from regions bearing a larger amount of information. This approach would guarantee that there would be sufficient remaining information to predict the missing patches of voxels. Given the entropy of the image, more patches are extracted from regions containing a larger amount of information. The size of the extracted patches varied from 3 × 3 to 25 × 25 mm^2^. Given the model trained for image inpainting, the trainable weights (layers) from self-learning were employed for the training of the target segmentation task (Fig. 2C). The last layer (Sigmoid) of the network in the self-learning model was replaced with a Softmax layer for the segmentation task. It should be noted that the trainable parameters were not frozen in the final target training to allow the model to fine-tune the parameters for the target segmentation task. The training of the model for object completion task, guided by the entropy of the input data, would lead to comprehensive and discriminative feature maps which would aid to boost the performance of the target segmentation task. A hybrid loss function based on a sum of Dice and cross-entropy loss was selected for the training of the target task, and the training of the proxy task (Fig. 2B) was performed based on a root mean square error (RMSE) loss function [12].

#### Deformable Data Augmentation

**Fig. 4:**
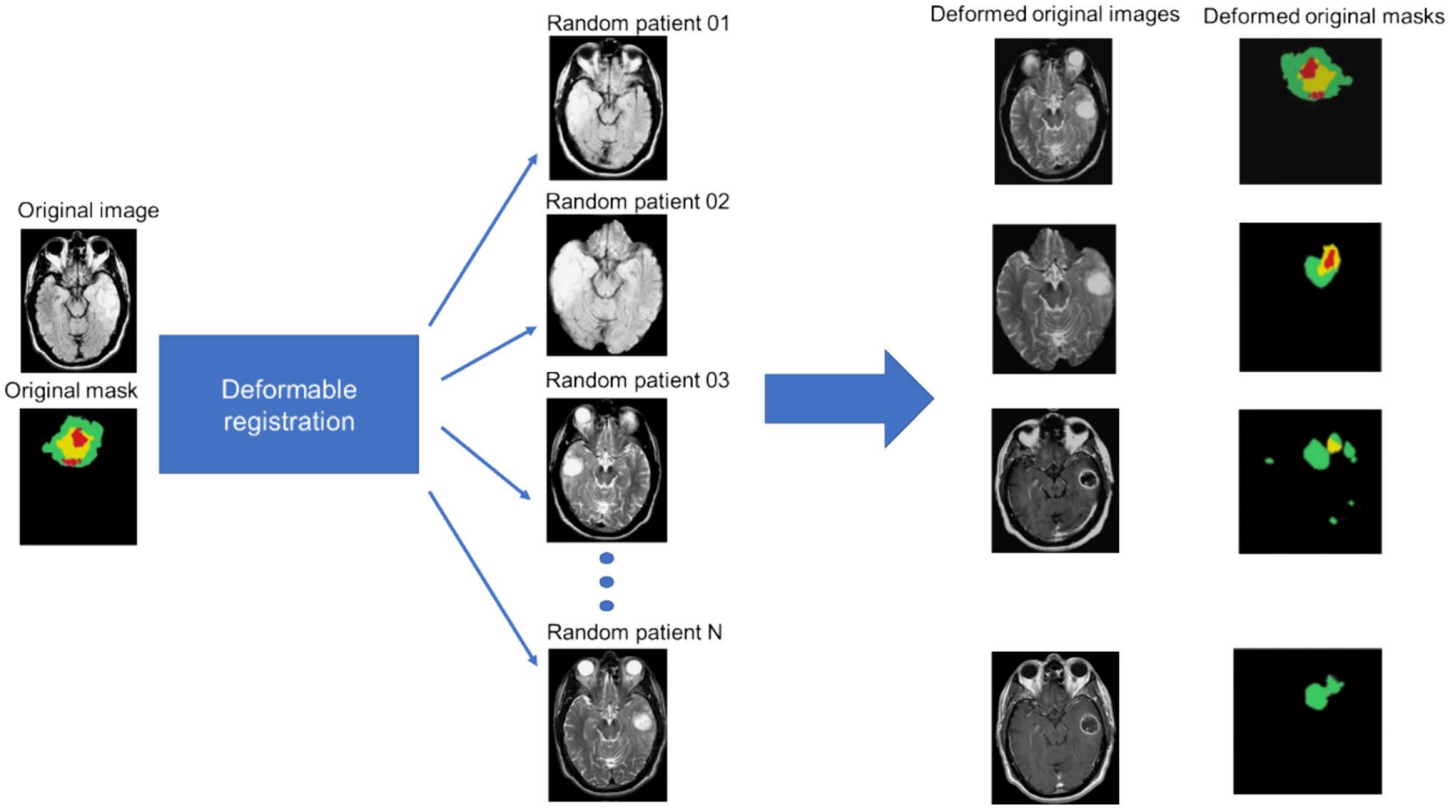
Illustration of the deformable data augmentation procedure

Contrastive learning and self-learning approaches are generally adopted to enhance the efficiency of model training. On the other hand, data augmentation techniques are able to enhance and add to the robustness of model training [5]. In this light, a deformable data augmentation technique is introduced to be evaluated next to the contrastive learning and self-learning approaches to provide a baseline for performance comparison. This data augmentation technique entails deformable registration of the original image (one of the subjects in the dataset) to the rest or several subjects in the dataset. In the first step, the original data is non-rigidly registered to the coordinate of the other subjects. These subjects are from clinical studies, involving realistic anatomical variations and poses, consequently leading to the generation of a realistic synthetic dataset (Fig. 4). Moreover, the registration subject should not be necessarily from the training dataset, wherein any clinical studies could be exploited in this registration process. Once the original image is deformed to the coordinate of the other subjects, the segmentation masks are identically transformed using the corresponding deformation map to create a new realistic synthetic training subject. Given a dataset with *n* subjects, this approach would generate *n*(*n − *1) synthetic datasets. This data augmentation technique was separately used for the training of the residual neural network model, illustrated in Fig. 2A without using any contrastive learning or self-learning. The Elastix package, developed in the ITK library, was employed to perform the deformable registration using a mutual information-based loss function.

#### Implementation Details

The implementation of contrastive and self-learning, along with the training and evaluation of deep learning models, was executed on 2-dimensional slices. However, for image registration to incorporate deformable data augmentation, a 3-dimensional approach was adopted. Subsequently, the final models underwent training and evaluation in 2D mode. The images in the three datasets were resampled to an isotropic voxel size of 1 mm. Notably, the training and evaluation procedures were conducted patient-wise, ensuring the entire dataset of each patient was exclusively utilized for either training or evaluation. This approach mitigated any potential correlation between different slices of the same patient to maintain data integrity and prevent model bias, ensuring robustness and generalizability.

For the kidney and hippocampus datasets, a fivefold cross-validation scheme (at each iteration, 42 and 52 subjects were kept as external test dataset, respectively) was adopted to evaluate the different training frameworks. For the BraTs dataset, 200 subjects were kept as external test dataset and the training of the models was performed on the remaining subjects. The contrastive learning reached its training loss after approximately 5 epochs when the learning rate was modified from 0.01 to 0.0005 following the recommendations made in [36]. The pretrained model in Fig. 1A was used for the training of the target task in Fig. 1B, wherein the trainable parameters were not frozen, and no specific restrictions were applied on the pretrained parameters. The learning rate for the training of the target task was set at 0.005 in the early epochs and reduced to 0.0001 in the later epochs. The training of the target task reached the loss plateau after 10 epochs. A batch size of 50 was set for training of the proxy and target tasks.

The training of the proxy task in the self-learning framework was performed based on an RMSE loss function using a batch size of 40 and learning rates varying from 0.005 to 0.0005. The training of the model (proxy task (Fig. 2B)) reached its loss plateau after 15 iterations. Similarly, the trainable parameters were not frozen from the proxy to target task training. The training of the target task in the self-learning procedure (Fig. 2C) was conducted using a batch size of 40 and learning rates varying from 0.005 to 0.0001, following the recommendations made in [36]. The target training (Fig. 2C) reached its loss plateau after about 10 epochs.

The training of the model using the deformable data augmentation was conducted similarly to the target training (Fig. 2C) in the self-learning framework. A batch size of 40 and learning rates varying from 0.01 to 0.0001 were selected for the training of the model. Approximately after 20 epochs, the model reached its peak performance based on a hybrid cross-entropy and Dice loss function.

### Evaluation Strategy

The evaluation of the different training frameworks, including contrastive learning, self-learning, and deformable data augmentation, was assessed using standard segmentation metrics. These include Jaccard (JC) (Eq. 2), Dice (Eq. 3), sensitivity (*S*) (Eq. 4), relative volume difference (RVD) (Eq. 5), Hausdorff distance (HD) (Eq. 6), and mean absolute surface distance (MASD) (Eq. 7).

2 $$JC(R,T)=\frac{\left|R\cap T\right|}{\left|R\cup T\right|}$$

3 $$Dice(Ref,T)=\frac{2\left|R\cap T\right|}{\left|R\right|+\left|T\right|}$$

4 $$S(Ref,T)=\frac{\left|R\cap T\right|}{\left|T\right|}$$

5 $$RVD(R,T)=100\times \frac{\left|T\right|-\left|R\right|}{\left|R\right|}$$

6 $$HD(R,T)=\underset{R}{\text{max}}\{\underset{T}{\text{min}}\{d\left(R,T\right)\}\}$$

7 $$MASD(R,T)=\frac{{d}_{ave } \left({S}_{R}, {S}_{T}\right) {+ d}_{ave } ({S}_{T}, {S}_{R})}{2}$$

Here, *R* denotes the reference binary mask and *T* indicates the estimated target structures from the input data. *d*_*ave*_*(S*_*R*_*,S*_*T*_*)* returns the average of distances (straight line) from all points on the reference surface (*S*_*R*_) to the estimated surface by the machine learning models (*S*_*T*_). The Hausdorff distance (HD) denotes the maximum distance between the surface of the reference mask (*R*) and the surface of the estimated structure by the machine (*T*).

Since the BraTS dataset contains more than 1000 subjects, the impact of the training dataset size on the performance of the different training frameworks was also investigated through training the model with 100, 150, 200, 250, 300, 350, 400, 450, 500, and 550 training samples. Moreover, to investigate the compound effect of self-learning and data augmentation, the residual network model pre-trained by the self-learning framework was fine-tuned for the target segmentation task using the deformable data augmentation scheme.

Statistically significant differences between the results of the different training frameworks was assessed through the paired t-test analysis, wherein a *p*-value of 0.05 was considered as threshold for statistical significance. Beyond assessing significance solely through p-values, confidence intervals (CIs) were computed between the various approaches using ANOVA analysis, augmented by Tukey’s honestly significant difference (HSD) post hoc test. This comprehensive approach aimed to further substantiate the significance of the observed differences among the approaches.

## Results

**Fig. 5:**
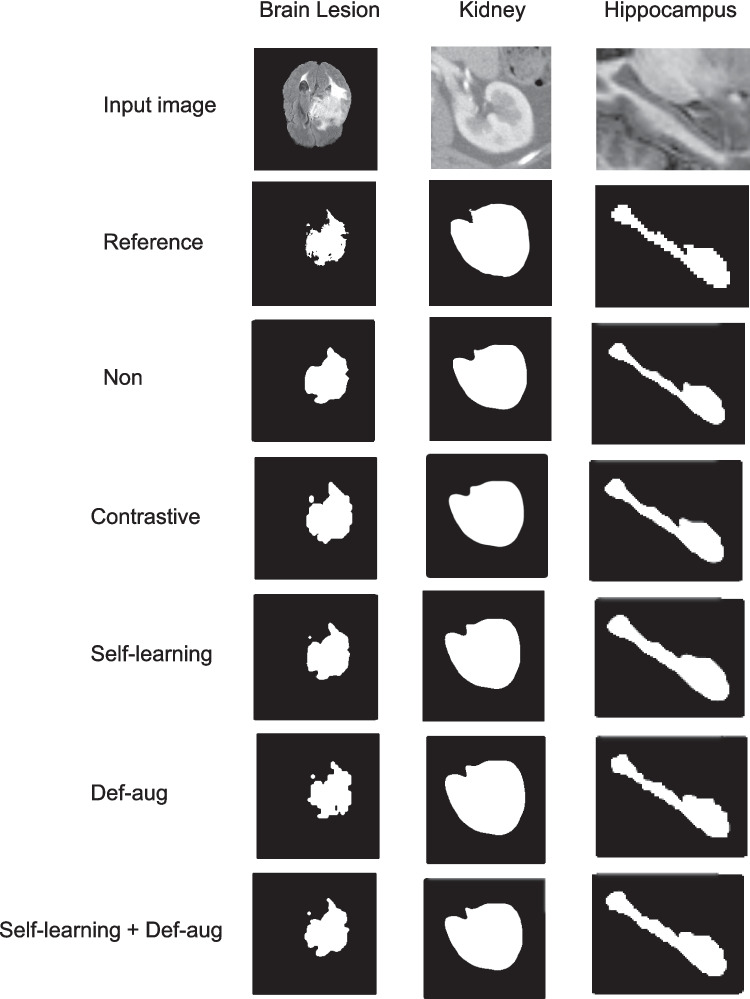
Representative outcome of different models for the kidney, hippocampus, and brain lesion segmentation tasks

Representative outcomes of different models for the kidney, hippocampus, and brain lesions are presented in Fig. 5. The results of seminal segmentation on the kidney and hippocampus datasets are reported in Tables 1 and 2 for the different model training strategies. The initial model training without using any self-learning (or contrastive learning) and data augmentation techniques is referred to as ‘Non.’ ‘Non’ refers to the ResNet model trained without any data augmentation, contrastive learning, or self-learning. The contrastive learning and self-learning techniques exhibited superior performance to the initial model in both datasets with statistically significant differences (Tables 3 and 4). Furthermore, the self-learning framework led to superior segmentation accuracy compared to contrastive learning for both kidney and hippocampus datasets (with significant *p*-values).

**Table 1:** Results of kidney segmentation from CT images using different model training frameworks

**Kidney**	**Dice**	**RVD (%)**	**JC**	***S***	**MASD (mm)**	**HD (mm)**
Non	0.868 ± 0.042	11.2 ± 1.9	0.871 ± 0.051	0.876 ± 0.034	3.81 ± 0.12	4.48 ± 0.27
Contrastive	0.871 ± 0.039	10.7 ± 1.8	0.882 ± 0.044	0.882 ± 0.030	2.70 ± 0.09	4.00 ± 0.25
Self-learning	0.913 ± 0.030	9.03 ± 1.4	0.902 ± 0.043	0.892 ± 0.026	1.90 ± 0.07	3.33 ± 0.19
Def-aug	0.920 ± 0.022	9.00 ± 1.4	0.905 ± 0.036	0.901 ± 0.023	1.85 ± 0.06	3.23 ± 0.18
Self-learning + Def-aug	0.920 ± 0.020	9.00 ± 1.3	0.906 ± 0.035	0.902 ± 0.023	1.85 ± 0.06	3.21 ± 0.18

**Table 2:** Results of hippocampus segmentation from MR images using different model training frameworks

**Hippo**	**Dice**	**RVD (%)**	**JC**	***S***	**MASD (mm)**	**HD (mm)**
Non	0.865 ± 0.048	9.0 ± 1.9	0.815 ± 0.023	0.806 ± 0.032	1.44 ± 0.10	4.10 ± 0.22
Contrastive	0.872 ± 0.045	8.1 ± 1.7	0.819 ± 0.020	0.810 ± 0.029	1.39 ± 0.09	4.00 ± 0.21
Self-learning	0.890 ± 0.035	7.2 ± 1.5	0.840 ± 0.017	0.831 ± 0.027	1.16 ± 0.08	2.34 ± 0.18
Def-aug	0.898 ± 0.027	6.9 ± 1.3	0.843 ± 0.015	0.835 ± 0.026	1.14 ± 0.08	2.33 ± 0.17
Self-learning + Def-aug	0.901 ± 0.024	6.8 ± 1.3	0.845 ± 0.014	0.837 ± 0.025	1.13 ± 0.08	2.33 ± 0.17

**Table 3:** *p*-values (confidence intervals (CIs)) were calculated between the results of the different training frameworks reported in Table 1

**Kidney**	**Dice**	**RVD (%)**	**JC**	***S***	**MASD (mm)**	**HD (mm)**
Non vs. contrastive	0.05 (0.001,0.005)	0.04 (0.29, 1.71)	0.03 0.001,0.020)	0.04 (0.006, 0.026)	0.02 (0.08, 1.20)	0.05 (0.007,0.830)
Contrastive vs. self-learning	0.02 (− 0.049, − 0.036)	0.02 (1.77, 2.234)	0.01 (0.005, 0.030)	0.04 (0.003,0.033)	0.03 (0.67, 1.09)	0.02 (0.546, 0.873)
Self-learning vs. Def-aug	0.04 (− 0.012, − 0.002)	0.06 (− 0.09, 0.96)	0.05 (0.008, 0.023)	0.05 (0.003, 0.016)	0.06 (− 0.01,0.29)	0.05 (0.056, 0.430)
Def-aug vs. self-learning + Def-aug	> 0.10 (− 0.004, 0.004)	> 0.10 (− 0.36, 0.36)	> 0.10 (− 0.001, 0.022)	> 0.10 (− 0.002, 0.002)	> 0.10 (− 0.01, 0.01)	0.10 (− 0.010, 0.070)

**Table 4:** *p*-values (confidence intervals (CIs)) were calculated between the results of the different training frameworks reported in Table 2

**Hippo**	**Dice**	**RVD (%)**	**JC**	***S***	**MASD (mm)**	**HD (mm)**
Non vs. contrastive	0.04 (0.012, 0.024)	0.03 (0.01, 1.06)	0.04 (0.001, 0.004)	0.05 (0.001, 0.008)	0.04 (0.01, 0.08)	0.05 (0.001, 0.262)
Contrastive vs. self-learning	0.01 (− 0.029, − 0.007)	0.01 (0.53, 1.07)	0.03 (0.006, 0.024)	0.04 (0.004, 0.025)	0.02 (0.21, 0.42)	< 0.01 1.01, 1.44)
Self-learning vs. Def-aug	0.05 (− 0.015, 0.017)	0.05 (0.01, 0.89)	0.06 (− 0.006, 0.018)	0.06 (− 0.007, 0.016)	0.04 (0.01, 0.25)	0.07 (− 0.022, 0.012)
Def-aug vs. self-learning + Def-aug	0.10 (− 0.005, 0.005)	> 0.10 (− 0.049, 0.39)	> 0.10 (− 0.005, 0.008)	> 0.10 (− 0.007, 0.008)	> 0.10 (− 0.01, 0.01)	> 0.10 (− 0.001, 0.001)

Deformable data augmentation technique and self-learning framework exhibited comparable results with barely significant differences (Tables 3 and 4). However, both techniques outperformed the initial model and the contrastive learning framework. The combination of self-learning and deformable data augmentation techniques did not lead to superior performance.

Evaluation of the models on the BraTS dataset demonstrated a similar trend, where contrastive learning exhibited superior performance to the initial model (the model trained without any self-learning or data augmentation) and inferior performance to the model trained with self-learning framework (Table 5). The differences between the contrastive and self-learning approaches were significant as reported in Table 6. Similar to the organ segmentation task, the self-learning and deformable data augmentation technique exhibited very comparable results with barely significant differences.

**Table 5:** Results of lesion segmentation from MR images using different model training frameworks

**BraTS**	**Dice**	**RVD (%)**	**JC**	***S***	**MASD (mm)**	**HD (mm)**
Non	0.860 ± 0.058	9.1 ± 120	0.813 ± 0.024	0.801 ± 0.033	1.46 ± 0.11	4.1 ± 0.23
Contrastive	0.870 ± 0.049	8.2 ± 1.8	0.817 ± 0.022	0.809 ± 0.030	1.40 ± 0.10	4.02 ± 0.21
Self-learning	0.891 ± 0.045	7.3 ± 1.6	0.837 ± 0.018	0.830 ± 0.027	1.17 ± 0.08	2.35 ± 0.19
Def-aug	0.897 ± 0.040	7.1 ± 1.3	0.839 ± 0.018	0.834 ± 0.025	1.15 ± 0.08	2.34 ± 0.18
Self-learning + Def-aug	0.899 ± 0.040	7.0 ± 1.3	0.838 ± 0.017	0.835 ± 0.025	1.15 ± 0.07	2.34 ± 0.17

**Table 6:** *p*-values (confidence intervals (CIs)) calculated between the results of the different training frameworks reported in Table 5

**BraTS**	**Dice**	**RVD (%)**	**JC**	***S***	**MASD (mm)**	**HD (mm)**
Non vs. contrastive	0.03 (0.001, 0.004)	0.03 (0.01, 0.90)	0.04 (0.001, 0.009)	0.05 (0.001, 0.010)	0.04 (0.01, 0.10)	0.04 (0.024, 0.175)
Contrastive vs. self-learning	0.03 (0.036, 0.009)	0.02 (0.34, 1.53)	0.04 (0.005, 0.025)	0.02 (0.002, 0.005)	0.02 (0.18, 0.22)	0.01 (1.020, 1.771)
Self-learning vs. Def-aug	0.05 (0.016, 0.020)	0.05 (0.01, 0.73)	0.07 (− 0.001, 0.001)	0.08 (− 0.007, 0.017)	0.04 (0.01, 0.07)	0.8 (− 0.139, 0.159)
Def-aug vs. self-learning + Def-aug	0.10 (− 0.010, 0.010)	> 0.10 (− 0.68, 0.48)	> 0.10 (− 0.001, 0.001)	> 0.10 (− 0.001, 0.004)	> 0.10 (− 0.03, 0.03)	> 0.1 (− 0.130, 0.130)

**Fig. 6:**
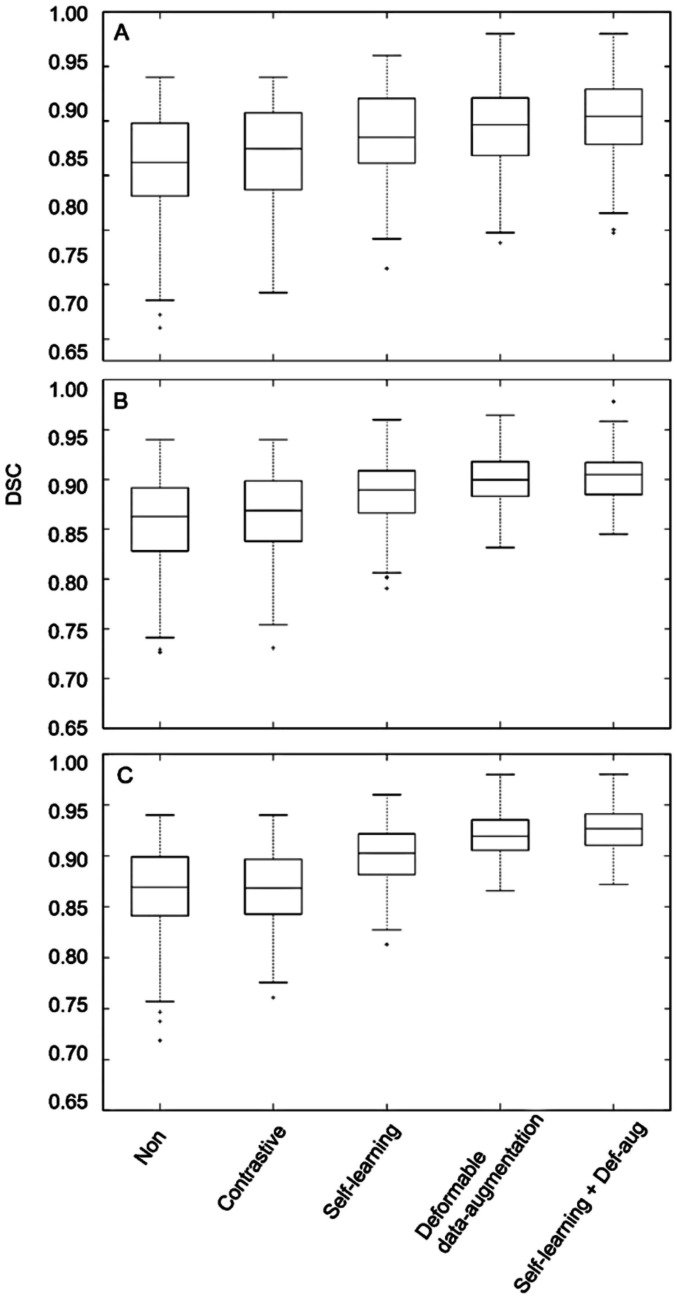
Box plots of Dice scores obtained from the different training frameworks for **A** BraTS, **B** hippocampus, and **C** kidney datasets

**Fig. 7:**
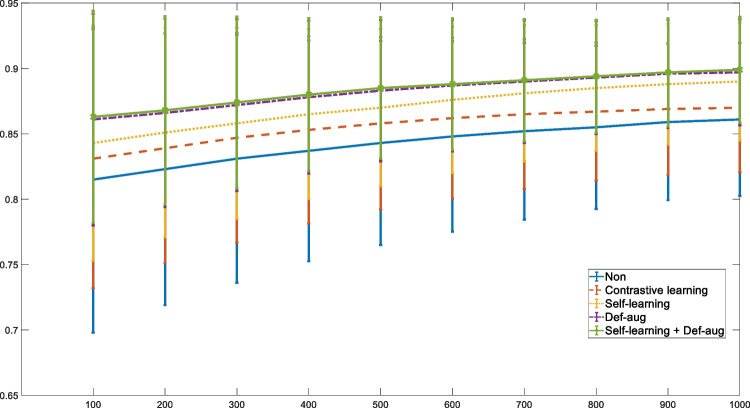
Dice scores for the varying training samples in the BraTS dataset obtained from the different approaches. The error bars indicate standard deviation

The box plots of the Dice scores obtained from the different models evaluated on the kidney, hippocampus, and BraTS datasets are illustrated in Fig. 6. A larger number of outlier or test samples with low scores were observed in the initial model compared to the self-learning and deformable data augmentation techniques. The impact of training dataset size on the performance of the different training frameworks was also investigated through training the model with 100, 150, 200, 250, 300, 350, 400, 450, 500, and 550 training samples. Figure 7 presents the Dice scores at different numbers of training samples, wherein smaller standard deviations are observed in self-learning and deformable data augmentation techniques.

## Discussion

Self-learning techniques are employed in the development of machine learning models to enhance the robustness and the overall performance of the models through the identification and extraction of more effective and discriminative features [37]. These techniques may play a critical role when the training samples are not sufficient/redundant, or the input data bears large intra- and/or inter-domain/center variations [18]. In these techniques, a proxy task is chosen to enrich the extracted feature maps, wherein the proxy task should provide a relevant/effective link to the target task [38]. In this work, contrastive learning, relying on patch discrimination as proxy task [19], and self-learning, relying on object completion as proxy task [39], were evaluated for the target tasks of lesion and organ segmentation from CT and MR images.

The self-learning techniques, enhanced with an effective object completion procedure, exhibited superior performance to contrastive learning on the three datasets. Although different network structures were exploited for the implementation of the contrastive learning (based on encoder-decoder modules) and self-learning (based on a residual network) techniques, these two networks exhibited very similar performance when evaluated without using contrastive learning or self-learning techniques. Object completion task requires extensive/comprehensive encoding of underlying patterns/structures from the input data which would greatly aid the segmentation task to discriminate the target structure from the background. The simple but effective patch extraction scheme, implemented in this work (Fig. 3), further improved the quality of the image completion task since a larger number of samples with adaptive sizes were taken from organs/lesions boundaries. Overall, the object completion, as a proxy task, would be beneficial for the target task of lesion or organ segmentation.

Since human anatomy follows an overall similar structure from one subject to another, deformable data augmentation technique could be employed for medical images [5], which might not be feasible for natural images. The deformable data augmentation technique would provide realistic new training samples that substantially differ from the original ones since they are non-linearly warped to another patient pose, anatomical structures, and dimensions. Deformable data augmentation was as much effective as the self-learning framework relying on the object completion task (the differences between these two models were barely significant). Conventional data augmentation technique, involving rotation, scaling, flipping, affine transform, etc., was also investigated, where inferior results were observed compared to the deformable data augmentation technique and self-learning framework.

In the literature, the best results for kidney segmentation were achieved using a 3D U-Net architecture, a convolutional neural network specifically designed for volumetric segmentation in biomedicine. This model attained a kidney Dice score of 0.974 and a tumor Dice score of 0.851, resulting in a composite score of 0.912 [40]. For hippocampus segmentation, several models, including nnU-Net, K.A.V. athlon, and Lupin, achieved an overall segmentation accuracy of 0.92 based on the Dice similarity coefficient (DSC) score [41]. In the brain tumor segmentation domain, a novel ensemble of multiple deep learning frameworks, namely, DeepSeg, nnU-Net, and DeepSCAN, secured the first place in the final evaluation on the BraTS testing dataset, with a Dice score of 0.9294 [42].

When there are sufficient training samples, the effectiveness of the contrastive and self-learning frameworks as well as data augmentation techniques might be limited [43]. In this light, the performance of these techniques was investigated on different sizes of the training dataset (Fig. 7) using the BraTS dataset. The self-learning and deformable data augmentation techniques substantially improved the performance of the machine learning models when smaller training sizes were examined (Fig. 7). In addition to overall improved Dice scores, lower standard deviations across the test subjects were observed when a combination of the self-learning and deformable data augmentation techniques were applied on small training samples. Moreover, no test samples with gross errors (outliers) were observed when self-learning and deformable data augmentation techniques (or a combination of them) were applied (Fig. 6). However, the original models (even the models trained with contrastive learning framework) led to gross errors for some test samples in each of the three datasets.

While this study offers valuable insights into enhancing deep learning model training with limited datasets through comparative analysis, several limitations merit consideration. The evaluation of data augmentation techniques, including deformable image alignment, alongside contrastive and self-learning approaches, focused primarily on CT and MR imaging modalities. However, the effectiveness of these methods may vary across different imaging modalities, such as PET, SPECT, and ultrasound, owing to distinct noise levels and structural information. Furthermore, the scope of this comparative study was constrained by the inclusion of only a few contrastive and self-learning methods, utilizing one or two deep learning architectures. Consequently, the generalizability of the findings may be limited, as the performance of these approaches could be influenced by factors not fully explored within the study’s framework.

## Conclusion

This work set out to compare the contrastive learning and self-learning frameworks as well as deformable data augmentation technique for the task of machine learning-based organ and lesion segmentation from medical images. The evaluation of these approaches for brain lesion and hippocampus segmentation from MR images, and kidney segmentation from CT images demonstrated comparable performance of the self-learning and deformable data augmentation techniques, outperforming the original model as well as the contrastive learning framework. The combination of the self-learning with deformable data augmentation led to a robust segmentation model with no outliers in the outcomes.
